# Miniaturized GaAs Nanowire Laser with a Metal Grating Reflector

**DOI:** 10.3390/nano10040680

**Published:** 2020-04-04

**Authors:** Wei Wei, Xin Yan, Xia Zhang

**Affiliations:** 1 School of Mechanical and Electric Engineering, Guangzhou University, Guangzhou 510006, China; 2 Photonics Research Centre, Department of Electronic and Information Engineering, The Hong Kong Polytechnic University, Hung Hom, Kowloon, Hong Kong, China; 3 State Key Laboratory of Information Photonics and Optical Communications, Beijing University of Posts and Telecommunications, Beijing 100876, China; xyan@bupt.edu.cn (X.Y.); xzhang@bupt.edu.cn (X.Z.)

**Keywords:** nanowire, nanolaser, metal grating

## Abstract

This work proposed a miniaturized nanowire laser with high end-facet reflection. The high end-facet reflection was realized by integrating an Ag grating between the nanowire and the substrate. Its propagation and reflection properties were calculated using the finite elements method. The simulation results show that the reflectivity can be as high as 77.6% for a nanowire diameter of 200 nm and a period of 20, which is nearly three times larger than that of the nanowire without a metal grating reflector. For an equal length of nanowire with/without the metal grating reflector, the corresponding threshold gain is approximately a quarter of that of the nanowire without the metal grating reflector. Owing to the high reflection, the length of the nanowire can be reduced to 0.9 μm for the period of 5, resulting in a genuine nanolaser, composed of nanowire, with three dimensions smaller than 1 μm (the diameter is 200 nm). The proposed nanowire laser with a lowered threshold and reduced dimensions would be of great significance in on-chip information systems and networks.

## 1. Introduction

Nanolasers hold the key position in on-chip sensing, optical interconnection and computing systems [1,2,3,4,5]. With cylindrical geometry and strong two-dimensional confinement of electrons, holes and photons, the independent semiconductor nanowire is one of the ideal candidates for nanolasers [6,7,8]. To date, room-temperature lasing emission has been realized in ZnO, GaN, CdS and GaAs nanowires, covering the optical spectrum from ultra-violet to near-infrared [9,10,11,12,13,14]. Among them, GaAs nanowires, with a direct band gap and high electron mobility, are considered to be a prime candidate for advanced opto-electronic devices [15]. However, due to the nanowires’ small size compared with their emission wavelength, ranging from 800 to 900 nm, the end facets of the nanowire display weak reflection at the near-infrared region, resulting in significant mirror losses [16]. Therefore, the light propagation in the nanowire has to be long enough to overcome a threshold loss induced by the weak facet reflectivities. The distinct index contrast reduces the diameter of the nanowires down to 200 nm, and even below the diffraction limit with the contribution of surface plasmons (collective electron oscillations) [17,18,19]. To keep the lasing emission from the nanowires at a relatively low threshold level, the nanowire’s length becomes too long compared with its diameter, which may impede its potential applications in nanophotonics and high-density photonics integration.

In this paper, an Ag grating with a short period number is proposed to enhance the end-facet reflection and lower the mirror loss of GaAs nanowires. The grating is employed as a high-quality reflector and placed at only one end facet to shrink the length of the nanowires. The other end-facet reflection is provided by the Au cap. The Ag grating and Au cap compose the optical resonant cavity. In that cavity, two mirrors of Ag grating and Au cap face each other, forming an optical resonator in which a light wave can be resonantly enhanced. To reveal the mechanism of the enhanced reflection by the short-period Ag grating, the finite elements method (FEM) is applied to a numerical simulation of the reflection and cavity properties. With the optimized structural parameters of the grating, the reflected spectrum can be located within the gain spectrum of the GaAs nanowires to generate an increase in feedback. Consequently, the lasing threshold will be lower than that without active feedback, and the nanowire’s length can be shortened while maintaining the laser’s power.

## 2. Structure of GaAs Nanowire with Ag Grating

**Figure 1:**
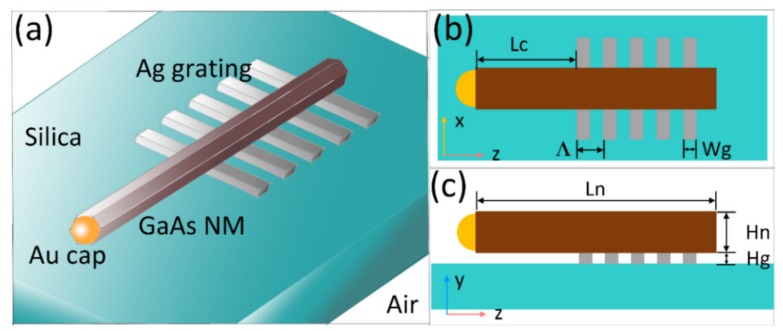
Schematic diagrams of the nanowire with the Ag grating reflector. 3D model (**a**), lateral view (**b**) and top view (**c**) of the proposed structure.

The schematic diagram of the GaAs nanowire with the Ag grating is shown in Figure 1. On the silica substrate there is an Ag grating, the permittivity of which is described by the Drude-Lorentz model

(1) $$\varepsilon \left(\omega \right)=1+\sum_{k}\frac{∆\varepsilon_{k}}{-\omega^{2}-a_{k}\left(i\omega \right)+b_{k}}$$

where $∆\varepsilon_{k}$, $a_{k}$ and $b_{k}$ are constants that provide the best fit for silver, when compared with the optical constant data of silver given by Palik et al. [20]. The terms $a_{k}$ and $b_{k}$ denote the damping frequency of the electron gas and the effective electron collision frequency, respectively. The Drude-Lorentz model is an extended Drude model, incorporating additional Lorentz terms [21,22,23]. Both of the models are classic models which describe the transport properties of electrons in metals. Figure 1 illustrates that Λ is the grating period; the width and height of the metal grating are denoted by $W_{g}$ and $H_{g}$, respectively. The duty cycle of the grating was fixed at 50% during the simulation. The GaAs nanowire is placed on the top of the grating. The part close to the dielectric interface is on the grating, to make the light propagated inside the nanowire interact with the metal grating. Meanwhile, the other part close to the Au cap is left suspended in the air. The Au cap on one top end of the nanowire is the Au particle, which is used as a catalyst during the growth of GaAs nanowires [14,24,25]. Its diameter is approximately equal to that of the nanowire. Due to the high reflection of the metal/dielectric interface, the Au cap is employed here as a high-quality reflector. Its reflectivity can be higher than 70%, depending on the nanowire’s diameter [13]. The Au cap naturally decreases the threshold and the nanowire’s length. The other end of the nanowire is cleaved, and the mirror is formed by the GaAs/air interface. The reflectivity of the dielectric interface for the fundamental mode is lower than 50%, and decreases with the shrinking nanowire diameter. It becomes less than 30% when the diameter is 200 nm. Therefore, only one end of the nanowire is located on the grating and the other end is left. Owing to the high modal confinement provided by the unique 1D geometry, the nanowire is employed both as the waveguide and gain medium. The Au cap and metal grating act as reflectors, forming the optical resonant cavity.

## 3. Results and Discussion

**Figure 2:**
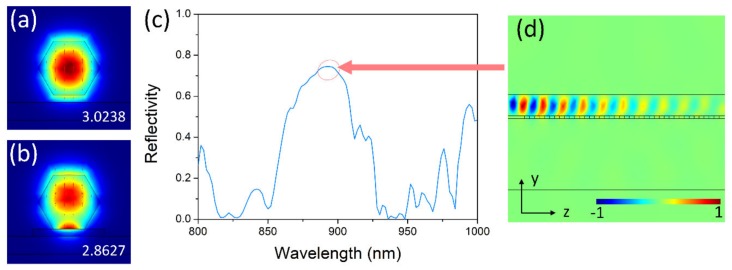
Modal profiles of *HE_11a_* for the nanowire in the air (**a**) and on the grating tooth (**b**). (**c**) Reflected spectrum of the grating. (**d**) Profile of *H*_z_ of the propagating mode. Numbers in (**a**) and (**b**) are modal effective indices. The pseudo-colors indicate the intensity of the magnetic field of *H*_z_.

When the nanowire is placed on the Ag grating, the light wave will be reflected by the grating, forming the gain feedback, which is shown in Figure 2. The large index difference of the waveguide mode *HE_11a_* for the nanowire in the air and that on the grating tooth enables the reflection of the light wave by the grating. The reflected spectrum of the grating is shown in Figure 2c, where the period and period number are 140 nm and 20, which makes the spectrum within the gain spectrum of the GaAs nanowire. The reflected spectrum has a wide bandwidth of 64 nm. Owing to the high index contrast, the grating, with a short period number of 20, can provide a maximum reflectivity of 74.5%. As shown in Figure 2d, the profile of *H*_z_ of the propagating mode is highly reflected by the Ag grating, and very little electromagnetic energy is transmitted through the nanowire.

**Figure 3:**
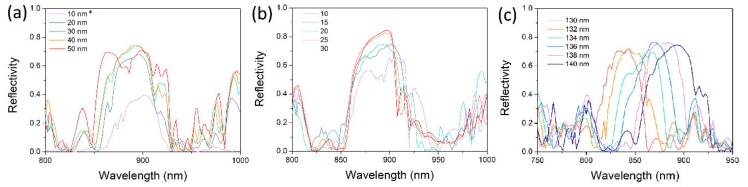
Reflected spectra of the grating for different grating heights (**a**), period numbers (**b**) and Λ (**c**). * Due to the limitation of classic model of Drude-Lorentz at the order of ~10 nm, the simulation result values may not be very accurate. The results given here are only to show the varying trend of reflection intensity as the grating height decreases.

The reflected spectrum, including bandwidth and peak, is dependent on the structural parameters of the grating. As depicted in Figure 3a, the grating height varies from 10 to 50 nm. The reflection is weakest for a grating height of 10 nm. It is significant to be note that due to the limitation of the classic Drude model and its extensions of the adopted Drude-Lorentz model at the order of ~10 nm, the simulation result values may not be very accurate [26]. The reflection curve of 10 nm here is only to demonstrate the varying trend as the grating height decreases. With the increasing height, the reflection becomes higher, and the bandwidth of the reflected spectrum becomes wider. However, reflection stays nearly similar when the height increases from 30 to 40 nm, and then becomes weaker at 50 nm. This can be attributed to the effective index contrast. The index contrast increases along with the height, resulting in higher reflection. However, when the grating height gets too large, the very high index contrast and the metal tooth are similar to a cliff or wall for the guided mode inside the nanowire, impeding the propagation and reflection of the mode. The period number is a direct factor in deciding the reflectivity of the grating. In Figure 3b, the reflection gets stronger with the increasing period number, from 10 to 30. When the period number increases from 25 to 30, the reflectivity increases by a very small amount. To be different from the dielectric grating with a small index contrast, the metal grating only needs a short period number to realize high reflection. The reflection capability of the grating gets saturated at a period number of 25. Furthermore, a long period number brings a long grating length, which also increases the nanowire’s cavity length. This is contradictory to the purpose of the integration with the metal grating. Thus, the period number would be 20 or 25. In the following calculation, all the period numbers are assumed to be 20. The grating period Λ, or width, is crucial to the peak of the reflected spectrum. As depicted in Figure 3c, the reflection peak displays red-shift with the increasing Λ By optimizing the period Λ or width, the reflected spectrum of the grating can be adjusted within, or to cover, the gain spectrum of the gain medium. The wavelength of the lasing emission from a GaAs nanowire most probably ranges from 850 to 880 nm [14]. Therefore, all of the structural parameters were selected to cover or partly cover that spectral region.

**Figure 4:**
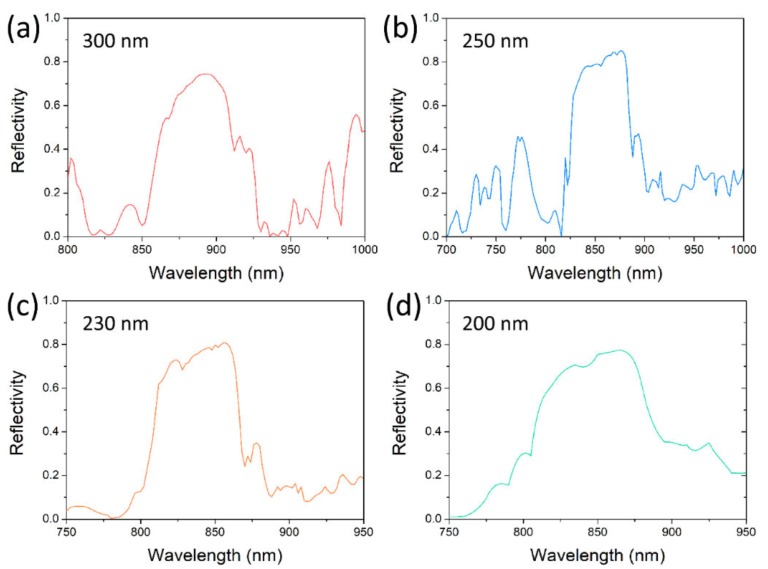
Reflected spectra for nanowire diameters of 300 nm (**a**), 250 nm (**b**), 230 nm (**c**) and 200 nm (**d**).

The photonic integration is going towards high density, requiring reduced dimensions of photonic components. As the nanowire diameter decreases to the limit of fundamental modes, the reflectivity decreases dramatically, together with the threshold gain. The metal grating reflector is more meaningful for nanowires with smaller diameters. The reflected spectra for nanowire diameters of 300 nm, 250 nm, 230 nm, and 200 nm are shown in Figure 4, where the other structural parameters of the grating height, duty cycle and period number are the same. The grating period or width are slightly tuned, to keep the spectra within the gain spectrum of the GaAs nanowire. The bandwidth and intensity of the reflection spectrum have negative relationships with the diameter, due to the index contrast increasing with the decreasing diameter. As the diameter decreases, the electromagnetic wave guided inside the nanowire has a more intense interaction with the metal grating tooth, resulting in a larger modal effective index, together with index contrast. 

**Figure 5:**
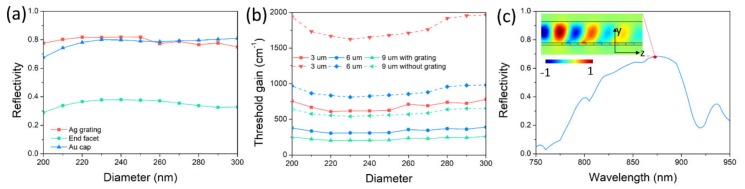
(**a**) Reflected spectra of the Ag grating, end facet and Au cap for nanowire diameter vary from 200 to 300 nm. (**b**) Threshold gain of nanowire with and without Ag grating for nanowire diameters varying from 200 to 300 nm. (**c**) Reflected spectrum for a period number of 5. Pseudo-colors indicate the intensity of the magnetic field of *H*_z_.

To compare the reflection between the Ag grating, end facet, and Au cap, functions of the Ag-grating reflectivity, end-facet reflectivity and Au-cap reflectivity on nanowire diameter are depicted in Figure 5a. The end-facet reflection is very weak and gets weaker for diameters of 200 nm and 220 nm. At a diameter of 200 nm, the end-facet reflectivity is below 30%. In contrast, for the Au-cap reflection, it is very strong even at a diameter of 200 nm. Its reflectivity can be 80% at larger diameters, and more than 65% at a diameter of 200 nm. Thus, the Au cap can provide excellent reflection for the propagated light wave with extremely small dimensions. This can be an additional advantage of the Au-catalyst growth method. For the reflection of the Ag grating, it is much stronger than the end-facet reflection. Its reflectivity is two times larger than its end-facet reflectivity. For small nanowire diameters below 260 nm, the reflectivity of Ag grating is even greater than Au-cap reflectivity. The introduction of the metal grating reflector is to compensate for the end-facet reflection, resulting in the reduced nanowire length or lowered threshold gain. The lasing threshold is the lowest excitation level, at which laser output is dominated by stimulated emission rather than spontaneous emission. The threshold gain $g_{th}$, which describes the required gain per unit length for lasing, is defined as [27]

(2) $$g_{th}=\frac{1}{\Gamma_{wg}}\left[\alpha_{i}+\frac{1}{L}\ln\left(\frac{1}{R}\right)\right]$$

where *R* denotes the geometric mean of the reflectivity of the end facets of the nanowire, and *L* is the length of the nanowire’s optical resonant cavity. $\Gamma_{wg}$ is the modal confinement factor, which is an indicator of how well the mode overlaps with the gain medium, and is defined as the ratio between the modal gain and material gain in the active region [28,29,30]

(3) $$\Gamma_{wg}=\frac{\frac{n_{a}}{2\eta_{0}}\int_{A_{a}}d\rho {\left|E\left(\rho \right)\right|}^{2}}{\int_{A_{a}}d\rho \frac{1}{2}Re\left[E\left(\rho \right)\times H^{*}\left(\rho \right)\right]\cdot \hat{z}}$$

where $\eta_{0}$ is the intrinsic impedance, $n_{a}$ is the index of the active region, $A_{a}$ is the cross section of the active region, A is the whole cross section ideally extending to infinity, and E and H are the complex electric and magnetic fields of the guided modes. The threshold gains of a nanowire laser with and without metal grating for diameters varying from 200 to 300 nm are shown in Figure 5b. Three lengths were chosen to demonstrate the threshold performance of the nanowire laser. As lasing emission is easily output from nanowires with lengths from 5–10 μm, lengths of 6 and 9 μm are typical parameters for a nanowire laser. A nanowire of 6 μm length without grating has a relatively high threshold gain, around 800 cm^−1^, and increases to about 1000 cm^−1^ when the diameter decreases to 200 nm. This is not beneficial for lasing emission from nanowire and requires high-power pump. If the length is changed to 9 μm, the threshold gain becomes moderate, ranging from 600 to 800 cm^−1^, which is helpful for lasing emission. However, for nanowire with metal grating, threshold gains for lengths of 6 and 9 μm are both low. The threshold gain of nanowire with a length of 6 μm ranges from 300 to 400 cm^−1^. For nanowire with a length of 9 μm, threshold gain can go down to 200 to 250 cm^−1^. At this threshold level, the laser can be easily lased without a high-power pump. To shrink the nanowire’s length, we make the length 3 μm, at which lasing emission requires a high-power pump. Its threshold gain ranges from 1600 to 2000 cm^−1^. With the metal grating, the threshold gain can go down to 600 to 800 cm^−1^, decreasing threshold gain by more than 2 times. At this threshold level, lasing is not hard to realize. Thus, the length of the nanowire laser can be shortened to 3 μm under moderate pump power. In high-density photonic integration, a nanolaser with three dimensions at nanoscale is preferred. Therefore, we make the length of the nanowire laser 900 nm by shortening the period number of the metal grating to 5. As shown in Figure 5c, the bandwidth becomes wider and the reflection becomes weaker than nanowire of 3 μm length. The maximum reflectivity decreases from 77.6% to 69.2%, and the corresponding threshold gain increases from 756 to 2978 cm^−1^. Lasing at a threshold gain of ~3000 cm^−1^ requires a high-power pump. Although the threshold gain is not low, a nanowire laser can be shortened to within 1 micron and is promising to lase under a high-power pump. In future research, if some additional techniques, like surface plasmons, were added onto the nanowire with metal grating, the confinement factor would be increased by more than 1.5 times, lowering the threshold gain further [31,32].

## 4. Conclusions

In summary, we proposed a metal grating with a short period to provide high reflection for the light wave propagating inside the nanowire. The metal grating is placed between the silica substrate and the nanowire. To shorten the nanowire’s length, the metal grating is only integrated with the end facet of the dielectric interface. The other end-facet reflection is provided by the Au cap, with a high reflectivity of around 70%. When the period number of the metal grating is set to 20, the grating can realize a maximum reflectivity of 81.8%. Moreover, a high reflectivity of 77.6% was realized for the nanowire diameter of 200 nm. The reflectivity is nearly three times larger than that without metal grating. Owing to the high reflection of the metal grating, the threshold gain of the nanowire laser can be decreased by more than 2.5 times. The length of the nanowire laser can be shortened to 3 μm under moderate pump power. With a high-power pump, the length of the nanowire laser could be shortened down to 900 nm, which is promising for the realization of lasing emission at a threshold gain of 2978 cm^−1^. Consequently, all of the three dimensions of the nanowire laser, especially the length, could be reduced below 1 micron. The proposed miniaturized nanowire laser with metal grating would be promising for use in on-chip sensing, optical interconnection and computing systems.
